# Titanium wear from magnetically controlled growing rods (MCGRs) for the treatment of spinal deformities in children

**DOI:** 10.1038/s41598-022-15057-1

**Published:** 2022-06-25

**Authors:** K. A. Lüders, L. Braunschweig, A. Zioła-Frankowska, A. Stojek, D. Jakkielska, A. Wichmann, G. H. Dihazi, F. Streit, S. E. Güsewell, T. C. Trüe, S. Lüders, J. Schlie, K. Tsaknakis, H. M. Lorenz, M. Frankowski, A. K. Hell

**Affiliations:** 1 Pediatric Orthopaedics, Department of Trauma, Orthopaedic and Plastic Surgery, University Medical Center Göttingen, Göttingen, Germany; 2 Faculty of Chemistry, Adam Mickiewicz University, Poznan, Poland; 3 Clinical Chemistry, University Medical Center Göttingen, Göttingen, Germany; 4 Mahr GmbH, Göttingen, Germany

**Keywords:** Diseases, Health care, Health occupations, Medical research, Risk factors, Chemistry

## Abstract

Magnetically controlled growing rods (MCGRs) are an effective treatment method for early-onset scoliosis (EOS). In recent years, increasing titanium wear was observed in tissue adjacent to implants and in blood samples of these patients. This study aims to investigate the potential correlation between amount of metal loss and titanium levels in blood during MCGR treatment as well as influencing factors for metal wear. In total, 44 MCGRs (*n* = 23 patients) were retrieved after an average of 2.6 years of implantation and analyzed using a tactile measurement instrument and subsequent metal loss calculation. Titanium plasma levels (*n* = 23) were obtained using inductively coupled plasma-mass spectrometry (ICP-MS). The correlation of both parameters as well as influencing factors were analyzed. Titanium abrasion on MCGRs was observed in the majority of implants. There was no correlation of metal implant wear or titanium plasma values to the duration of MCGR implantation time, number of external lengthening procedures, patient’s ambulatory status, gender, weight or height. Material loss on the MCGRs showed a positive correlation to titanium blood plasma values. The present study is one of the first studies to analyze retrieved MCGRs using high-precision metrological techniques and compare these results with ICP-MS analyses determining blood titanium values.

## Introduction

Children with progressive early-onset scoliosis (EOS) often require surgical treatment to control the deformity, to preserve the growth potential of the spine and to prevent thoracic insufficiency syndrome^1^. During the last decades, several growth-friendly spinal implants have been introduced, most of them requiring repetitive surgical interventions for implant lengthening to preserve spinal growth. Recently, magnetically controlled growing rods (MCGRs) render multiple surgical lengthening procedures obsolete in a subgroup of EOS patients^2^. A number of studies have reported the results on the short- and long-term outcome of MCGRs in the management of early spinal deformity^3–6^. While the ability to control deformity progression and the benefit of outpatient distractions of MCGRs is evident, concerns remain about the risk of complications including generation of excessive metal debris.

MCGRs consist of a biomedical titanium alloy (Ti-6Al-4V)^7^. Despite biological and mechanical compatibility of titanium, microscopic metal particles have been observed extensively within the rods^8^ and in the surrounding tissue of the rods^7,9–11^. It has been suggested that titanium wear debris can locally lead to inflammatory reactions, osteolysis or implant loosening^11–13^. Additionally, it is thought that titanium particles enter the blood circulation and accumulate in other tissues and distant organs^14^. With the ability to generate reactive oxygen species, titanium can induce oxidative stress and may increase the risk of cancer^15^. These possible long-term consequences of implant-caused titanium particles have gained attention and raised concerns. Several studies showed increased titanium concentrations in the patient’s whole blood, serum or urine after implantation of spinal titanium devices in children^9,10^.

Also wearing marks as a sign of metal abrasion from MCGRs have been observed^8,16,17^, but only few studies have used metrological techniques to characterize the extent of metal abrasion^16^. Measuring abrasion from implants is challenging, especially due to the small scale of changes on the material and the individual shapes in which abrasion may display. Thus, very precise instrumentation that can detect abrasion in the occurring shapes is necessary.

The aim of this study was to investigate the extent of titanium wear (i.e. volume of abrased material) in children during MCGR treatment and the potential correlation between material loss on the MCGRs and titanium values measured in blood plasma. Furthermore, patient-related factors (weight, height, gender, ambulatory ability) and implant-related factors (duration of treatment, number of distraction procedures) that possibly affect material loss on the rods were evaluated.

## Material and methods

**Figure 1:**
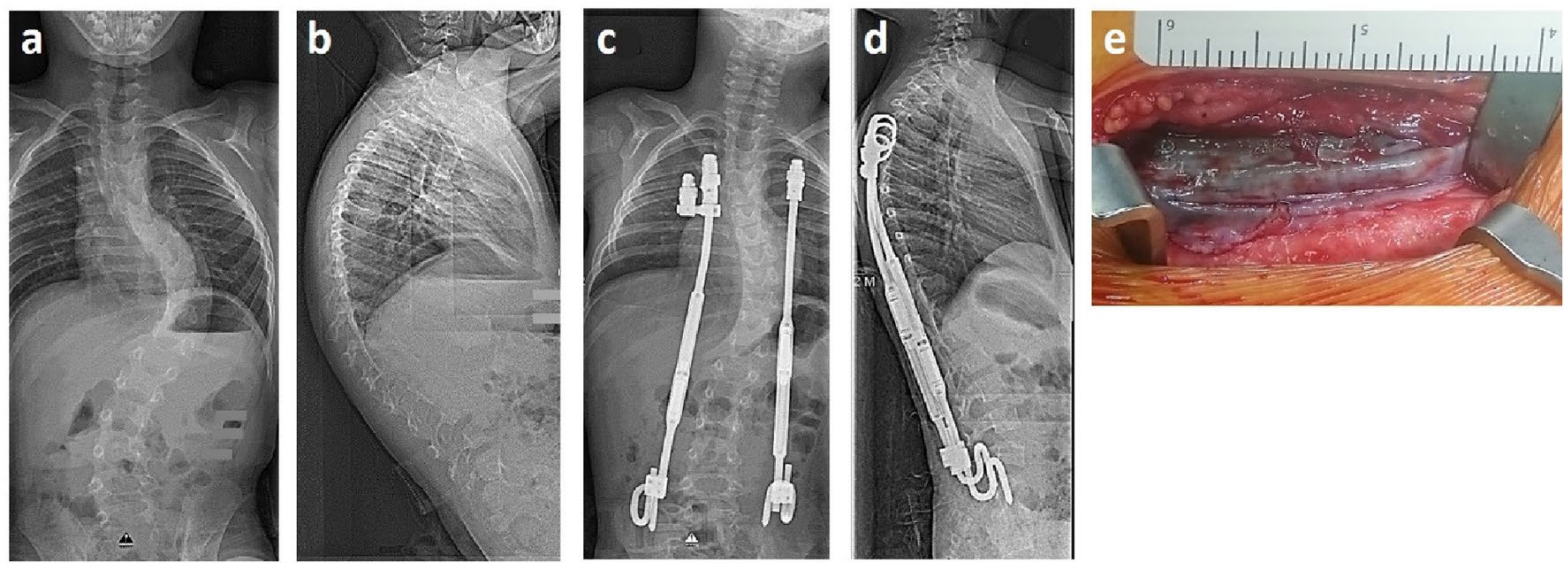
Bilateral MCGR system for spinal deformity correction in children. Anterior–posterior (**a**,**c**) and lateral radiographs (**b**,**d**) of a 6-year old boy with neuromuscular scoliosis. The main scoliotic curve was corrected from 71° (**a**) to 32° (**c**) with additional improvement of the sagittal profile (**b**,**d**). Intraoperatively, discoloration of the adjacent soft tissue was found (**e**).

After approval of the institutional ethical review committee, we performed a prospective study on 23 children (average age 10.7 years, range 7.4–14.9) diagnosed with scoliosis (neuromuscular *n* = 19, congenital *n* = 2, idiopathic *n* = 1, syndromic *n* = 1), who were treated with paraspinal MCGRs with a rib cradle and pelvic hook fixation with one standard and one off-set implant (Fig. 1). All participants were instructed about the purpose of the study and oral and written informed consent was obtained from all subjects and their legal guardians. After implantation of the MCGRs, repetitive standardized outpatient distraction procedures of 5 mm per rod were carried out every 3 months. After maximal extension and/or complications requiring surgery, surgical MCGR exchange was performed after an average of 2.6 years. During surgery, patient blood samples were acquired and explanted MCGRs were harvested. We did not observe any technical difficulty with revisions owing to metallosis. Patient demographic and clinical data were sampled for correlation to titanium abrasion and blood levels.

### Plasma analysis

After induction of anesthesia and before starting the exchange or revision surgery, 2–5 mL whole blood of each patient (*n* = 23) was collected in EDTA-monovettes (Sarstedt AG & Co. KG, Germany). Whole blood samples of control group patients (*n* = 9) were collected after anesthesia accordingly. Centrifugation for 10 min at 2000×*g* at room temperature separated the cells from the plasma. The supernatant designated as plasma was transferred and aliquoted in clean tubes and immediately preserved at − 20 °C.

An inductively coupled plasma-mass spectrometer (ICP-MS) model 2030 (Shimadzu, Japan) was used to assay blood plasma for titanium (Table 1).

**Table 1:** ICP-MS 2030 (Shimadzu, Japan) measurement parameters.

Parameter and accessories	Value
Radio frequency power generator	1.2 kW
Gas type	Argon
Plasma gas flow rate	8.0 L/min
Auxiliary gas flow rate	1.1 L/min
Nebulization gas flow rate	0.7 L/min
Torch	Mini-torch (quartz)
Nebulizer	Coaxial
Spray chamber temperature	3 °C
Drain	Gravity fed
Internal standard	Automatic addition
Sampling depth	7 mm
Collision cell gas flow (He)	4.0 mL/min
Cell voltage	− 35 V
Energy filter	5.0 V
Number of replicates	3
Integration conditions/number of scans	10
Integration time	2 s

Serial dilutions of ICP-MS single titanium standard solutions were used for calibration (Sigma Aldrich Merck group, Poland). Additionally, for ICP-MS Sc in 1% HNO_3_ ≥ 99.999% trace metals basis (Sigma Aldrich Merck group, Poland) was used as internal standards (automatically added during analysis through T-piece). Deionized water was obtained from the Milli-Q Direct 8 Water Purification System (Merck Millipore) and applied for sample (pre) treatment and dilutions. A series of spikes samples (S1 Human Serum, Normal, BCR637 and S7023 as a matrix simulation samples) were prepared and analyzed to validate the sample preparation method (no CRM and SRM are available for Ti analysis). Analysis was in the range of recovery from 86 to 110%. The plasma samples were prepared using the method of additions 100 µl sample + 300 µl HNO_3_ (70%, purified by redistillation, Sigma Aldrich, Poland) and pretreatment in a 60 °C on water bath for 2 h. The detection limit for titanium was 0.2 ng/mL. Any concentrations below this limit of detection were assigned as zero.

### Analysis of MCGRs

**Figure 2:**
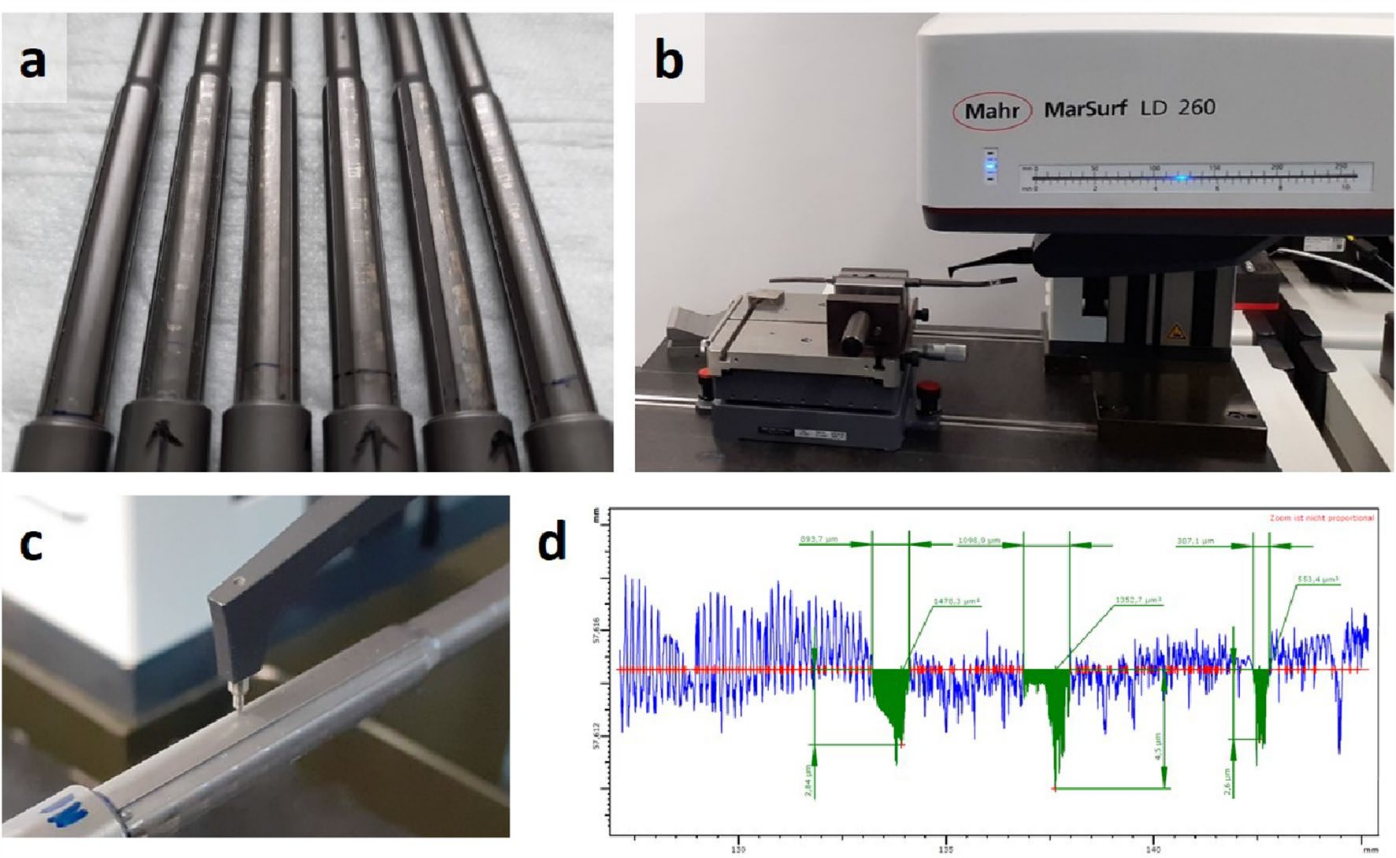
Tactile measurement of MCGRs. Selection of MCGRs with completely expanded inner segments, displaying one rod without detectable abrasion (left rod) and five rods with metal abrasion (**a**). Display of an individual MCGR fixed within the MarSurf LD260 machine (Mahr GmbH, Germany) with one segment facing upwards at a time (**b**). The diamond-tip of the probe arm was set to record longitudinal tactile traces along this segment of the MCGR (**c**). Screenshot of the software MarWin (Mahr GmbH, Germany), which was used to measure the depth, width and area of individual notches, representing metal abrasion along the tactile traces (**d**).

**Figure 3:**
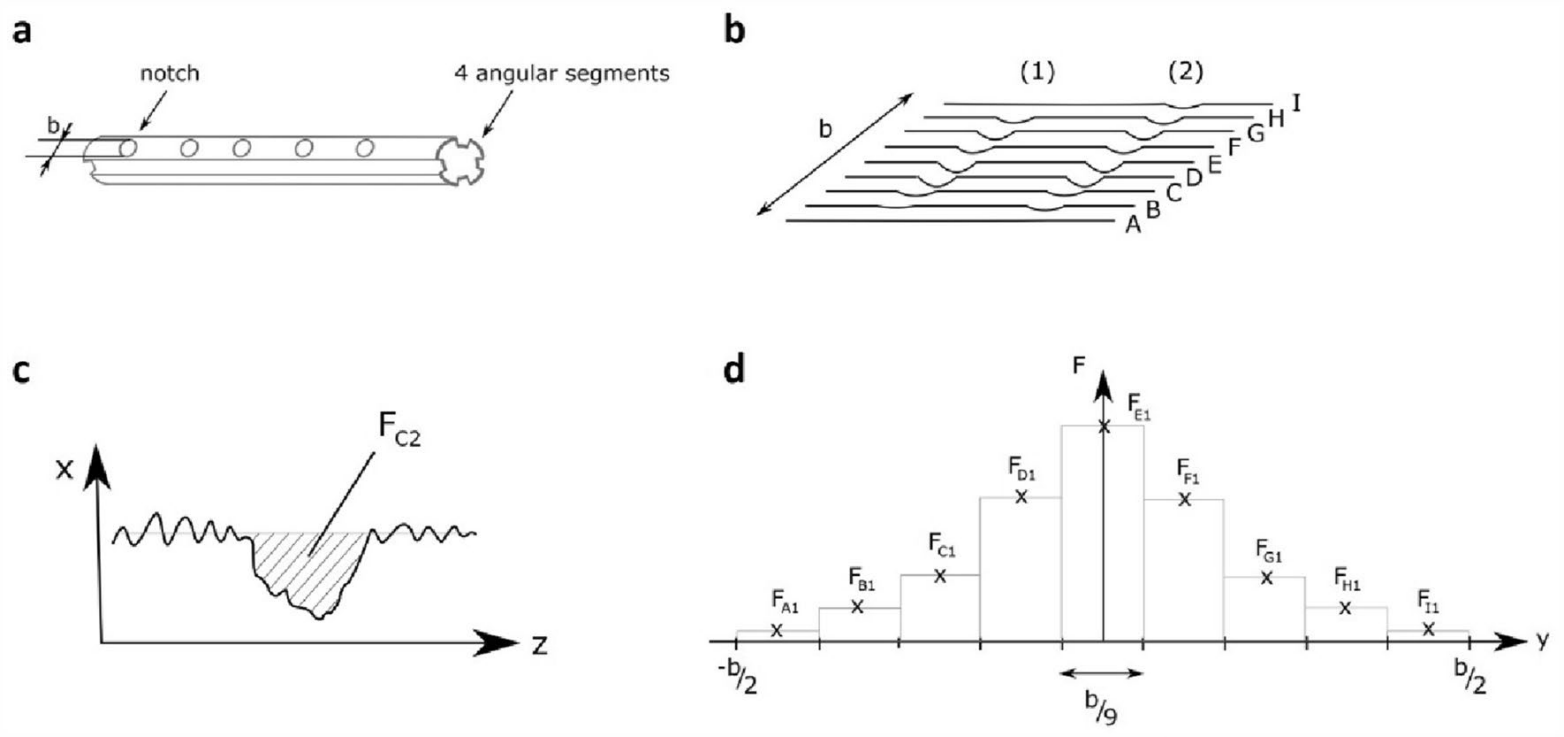
Volume of abrased material calculated from tactile traces. Schematic representation of the inner segment of a MCGR with five representative notches on one angular segment; with b being the width of the segment (**a**). Nine longitudinal traces (A–I) with a distance of 0.5 mm from each other were recorded longitudinally along the segment, so that the entire width b (approximately 5 mm) of the segment was covered by regular traces. In (**b**) two representative notches (1) and (2) are shown. Schematic representation of a notch as displayed in the software MarWin. Using the software, the 2D-area of each individual notch (here F_C2_) on each individual trace was determined (**c**). The volume was calculated from the areas by numerical integration. Therefore, the area of each notch on a trace (e.g. F_A1_, F_B1_ etc.) was multiplied by 0.5 mm, which is the distance between the traces (**d**).

In total, 44 implants (NuVasive, USA) were retrieved from 23 patients (21 patients with bilateral and two patients with unilateral implant systems). After explantation, MCGRs were cleaned with H_2_O_2_. If not yet completely expanded, MCGR were magnetically expanded to their maximum length, so that the inner part of the rod, which is located in a cylinder in the retracted condition, became accessible. On this inner part, consisting of four segments (Fig. 3a), obvious traces of metal wear were visible (Fig. 2a). To determine the volume of metal loss, all angular segments with visible metal wear were measured using a tactile measurement instrument, the MarSurf LD260 (Mahr GmbH, Germany) (Fig. 2b,c). A probe arm with a diamond-tip measured tactile traces longitudinally along the segments. The speed was set at 0.5 mm/s, resolution in z was 0.8 nm and the tactile force was 0.75 mN (recommended tactile force for roughness measurements after ISO 3274). The measuring point density was set at 4.000 measuring points per mm (one measuring point every 0.25 µm). The traces were analyzed using the software MarWin v.11.00-19 (Mahr GmbH, Germany) (Fig. 2d) and the volume of abrased material was calculated (Fig. 3).

In order to calculate the volume of abrased material, nine parallel tactile traces with a distance of 0.5 mm to each other were recorded longitudinally along a segment, so that the entire width of the segment of approximately 5 mm was covered with regular tactile traces. Performing pre-tests, we determined that nine traces with a distance of 0.5 mm to each other were suitable to portray the surface with a good coverage area of the rod, while keeping the measurement time reasonable (approximately 15 min per segment). The Software MarWin was used to calculate the 2D-area of each notch on a trace, with notches representing abrased material. The 3D-volume of abrased material was calculated by numerical integration (i.e. multiplying the 2D-areas by 0.5 mm) (Fig. 3). The accuracy of the measurement was estimated using error propagation based on the technical data of the measuring device and the described evaluation procedure. The calculated uncertainty is about 15% of the estimated values.

The obtained data were reviewed statistically using GraphPad Prism (Microsoft Corporations, USA). All data are presented as mean ± standard deviation. Statistical significance was determined with *p* < 0.05 (*).

### Ethics approval and consent to participate

All procedures performed in studies involving human participants were in accordance with the ethical standards of the institutional and/or national research committee and with the 1964 Helsinki declaration and its later amendments or comparable ethical standards. All participants were informed about the purpose of the study and oral and written informed consent was obtained. The Institutional Ethics Committee of University Medical Center Göttingen approved the study (reference number 33/8/17).

## Results

Data of 23 scoliotic children (14 females, 9 males) with progressive scoliosis and implanted MCGRs were analyzed (Table 2). Surgical implantation of the initial rods was carried out at a mean age of 6.4 years (range 1.8–10.9). Rod analysis was performed using the initial MCGRs in twelve cases, whereas eleven patients had implant exchange before MCGR device analysis was performed. All analyzed rods were implanted between 2015 and 2019. Therefore, the newest possible implant generation was used. Implant revision and subsequent MCGR device analysis were performed after 2.6 years (range 0.8–3.4) due to maximum extension of the MCGRs (*n* = 20) or a required complication-related revision surgery (*n* = 3). Complications requiring revisions occurred in three patients and were implant failure (breakage of the pelvic hook *n* = 1, dislocation of the rod *n* = 1) or implant infection (*n* = 1). All except one rod were functioning at removal. End of skeletal growth was reached in four cases and thus, spinal fusion was performed some weeks after MCGR removal, whereas 16 patients received a new MCGR at the surgery of removal of the maximally expanded rod. MCGR revision and blood sampling of all participating patients (*n* = 23) was done at an average age of 10.7 years (range 7.4–14.9). At that time point, patients had been treated with MCGRs for 4.2 years (range 0.9–10.7) on average. No apparent clinical symptoms or complaints related to metallosis were observed. Plasma samples from nine control scoliotic children with no implants so far at average age of 7.7 years (range 4.8–13.8) were analyzed in accordance with the patients’ samples.

**Table 2:** Patient demographics.

Variable	Value
Number of patients	23
Age analysis (years)	10.7 ± 2.3 (range 7.4–14.9)
Number of patients without prior implant exchange at time of analysis	12
Number of patients with prior implant exchange at time of analysis	11
**Analysis of metal loss on MCGRs (*n*** **=** **23)**	
Age implantation of analyzed MCGR (years)	8.1 ± 2.2 (range 4.7–12.1)
Follow-up until implant exchange surgery (years)	2.6 ± 0.7 (range 0.8–3.4)
Age at MCGR revision (years)	10.7 ± 2.3 (range 7.4–14.9)
**Analysis of plasma samples (*n* = 23)**	
Age implantation of initial MCGR (years)	6.4 ± 2.5 (range 1.8–10.9)
Follow-up with MCGR until blood sampling (years)	4.2 ± 2.1 (range 0.9–10.7)
Age at blood sampling (years)	10.7 ± 2.3 (range 7.4–14.9)
Number of implants	1.7 (range 1–5)
Control children (*n* = 9): age at blood sampling (years)	7.7 ± 2.5 (range 4.8–13.8)

**Figure 4:**
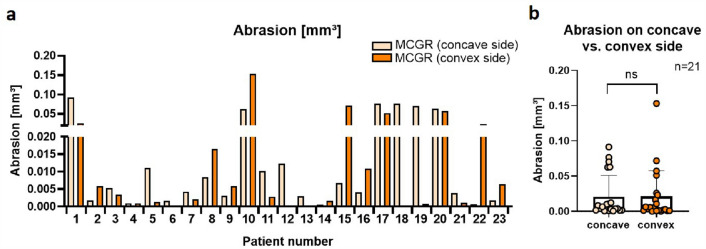
Titanium abrasion in mm^3^ for all measured rods of the 23 patients. 21 patients had two rods implanted (concave, convex), whereas two patients (patient number 13 and 18) only had one implant on the concave side. The convex rods of patient 6 and 12 displayed no and very little abrasion (0.00016 mm^3^) respectively, while patient 10 showed maximum values (**a**). No significant difference was detected between abrasion of MCGRs implanted on the concave or convex side of the scoliotic spine (paired *t* test; *p* = 0.9913) (**b**).

Measurement of abrasion on the 44 explanted MCGRs of the 23 patients showed metal loss on all except for one MCGR (97.7%). Metal loss was measurable on one out of the four segments in 30 rods (68.2%), two segments in 10 rods (22.7%) and three segments in 4 rods (9.1%). The volume of abrased material per rod was > 0.001 mm^3^ in 37 of 44 cases (84%), > 0.002 mm^3^ in 31 of 44 cases (70.5%), > 0.01 mm^3^ in 17 cases (38.6%), > 0.05 mm^3^ in 10 cases (22.7%) and > 0.1 mm^3^ (0.15 mm^3^) in one case (2.3%). No difference between MCGRs on the concave and convex side of the scoliotic curve could be observed (Fig. 4). The extent of implant abrasion did not correlate with the time of MCGR implantation, number of elongation procedures, patient’s ambulatory status, gender, weight or height (data not shown).

**Figure 5:**
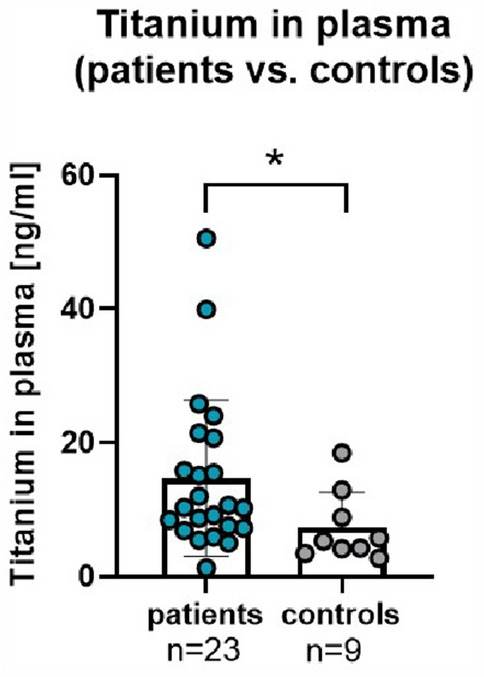
Titanium plasma analysis. Significant difference in titanium wear in MCGR-patients (*n* = 23) in comparison to age-matched controls (*n* = 9; *p* = 0.020). Unpaired *t* test with Welch’s correction (different SD assumed).

To evaluate a potential correlation of measured metal loss on the MCGR implants to titanium plasma values, blood samples were taken during the MCGRs exchange or explantation surgery and these were also compared to healthy controls (*n* = 9). Titanium values were significantly different (*p* = 0.020) when comparing patients (averaged 14.7 ± 11.4 ng/mL) to age-matched healthy individuals (7.4 ± 4.9 ng/mL) (Fig. 5).

Plasma titanium values did not correlate with the time of MCGR implantation (the measured implant time as well as the overall time), number of elongation procedures, patient’s ambulatory status, gender, weight or height (data not shown).

**Figure 6:**
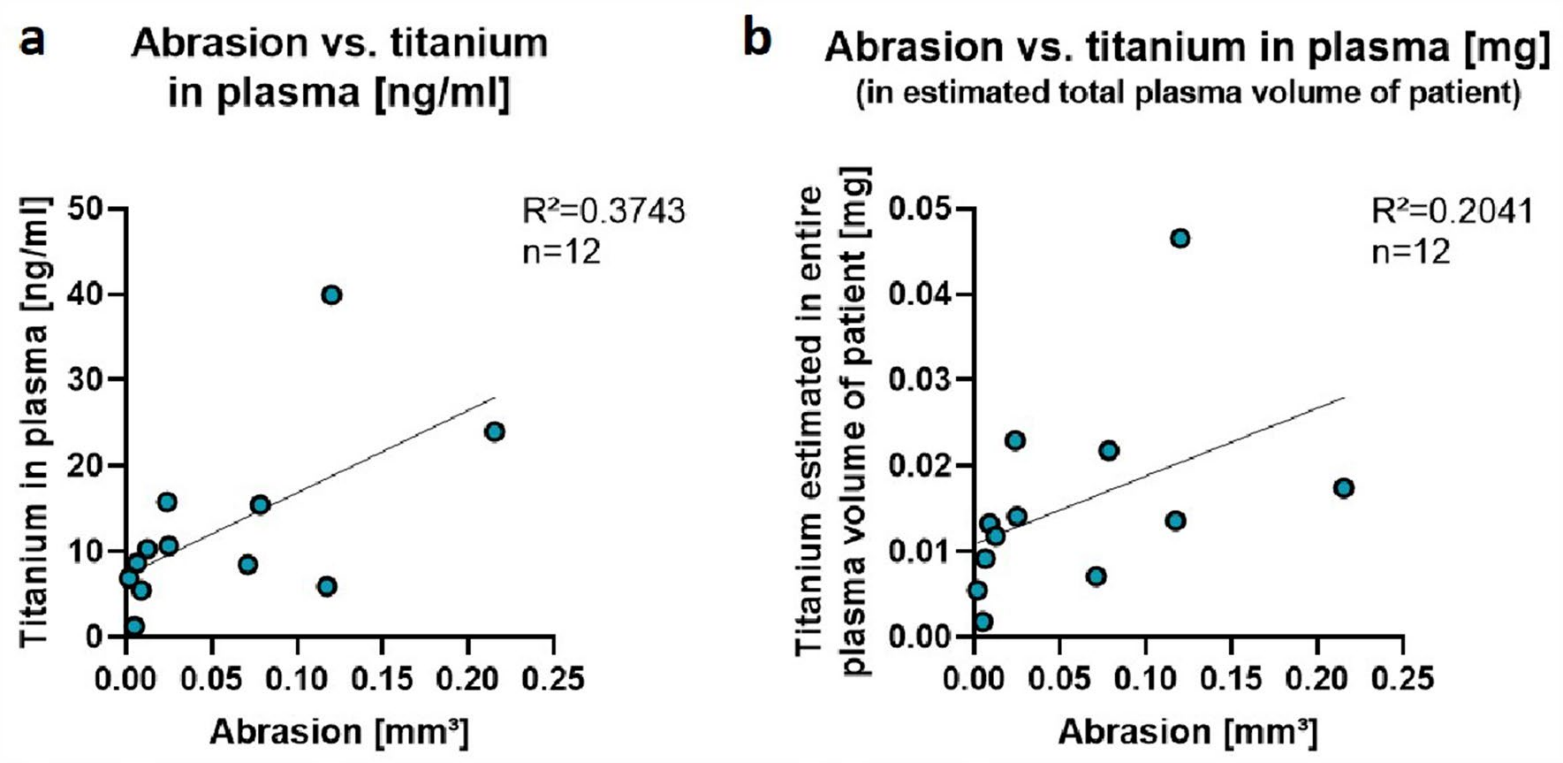
Correlation of abrasion and titanium analysis in plasma. For patients, whose implants at the time of blood sampling were the initial ones (*n* = 12), linear regression analysis between abrasion on implants in mm^3^ (x-axis) and titanium in plasma samples in ng/mL (y-axis) display a positive correlation (R^2^ = 0.3743; deviation of slope from zero significant *p* = 0.0345) (**a**). When extrapolating the measured titanium concentrations to the estimated entire plasma volume of each patient, the positive relationship remained (R^2^ = 0.2041; deviation of slope from zero not significant *p* = 0.1404) (**b**).

To determine whether metal loss on the rods correlates with blood titanium, analysis was only performed of those patients, whose implants at the time of blood sampling were the initial ones (*n* = 12). Linear regression analysis showed a positive correlation between both titanium wear measurements (abrasion on implants and titanium particles in blood samples) with R^2^ = 0.3743 and a none-negligible scattering (Fig. 6a). Additionally, we extrapolated the plasma titanium value of each patient to the blood volume to exclude bias due to high variance in age, weight and therefore blood volume of the participants. Blood volume was estimated by 80 mL/kg body weight and ranged from 1.4 to 4.2 L. We assumed the plasma to be 55% of the total blood and expressed the value in mg titanium in total estimated plasma volume for each patient. A positive relationship between the abrasion method and this extrapolated titanium value in plasma remained with R^2^ = 0.2041 (Fig. 6b).

## Discussion

MCGR devices have become a preferred treatment option for children with severe progressive spinal deformity^2^, thus avoiding repetitive surgical implant lengthening. Efficiency of these implants could be proven in several studies^3–5^, however, implant related complications still remain. MCGRs are usually implanted for several years during the critical pediatric growth period, therefore, the extent of metal wear and the potential hazards of titanium in the pediatric organism gains importance for the evaluation of the current standard treatment of EOS.

The presented data report on titanium wear analysis of 23 pediatric patients with scoliosis both in blood samples and on the explanted MCGR implant itself. Three main observations could be found: Firstly, titanium abrasion was observed in the majority of analyzed rods. Secondly, duration of MCGR implantation time, number of external lengthening procedures, patient’s ambulatory status, gender, weight or height did not influence metal wear or titanium plasma values. Thirdly, material loss on the MCGRs showed a positive correlation to titanium blood plasma values.

Other studies could find titanium wear debris inside the rods for all observed cases^8^. It is suggested that off axis loading causes the extending bar of the rod to contact on the internal surface of the rod housing^8^. Obvious metallosis in the surrounding tissue of the implants can be explained either by growth marks on the extending bar of the rod as a result of high stress of the rods during the lengthening procedures^18^ or by leakage of titanium wear from inside the rod^8^. We could find a certain correlation between extent of metal loss on the rods and measured titanium values in the blood. However, blood plasma titanium values do not reflect local titanium debris in the surrounding tissue or titanium that may have been deposited in organs or excreted. Therefore, a direct correlation to the overall titanium content in the pediatric body cannot be made from the presented data.

In the present study, patients showed on average twice as high values of blood titanium compared to controls without a device implanted (with a high variance among individual patients). Other studies could also detect increased blood titanium values after implantation of titanium spinal implants. Li et al. determined three times higher values for patients with MCGRs (4.5 ng/mL) than controls (1.5 ng/mL)^19^. Yilgor et al. found four times higher values of titanium in patients with MCGRs (10.2 ng/mL) than in controls (2.8 ng/mL)^7^. Borde et al. found even higher values (15.9 ng/mL) for patients with MCGRs^20^. Therefore, our measured values of 14.7 ng/mL are in the range of the literature values.

In the control group, certain titanium plasma values were detected in some individuals, probably due to exposure to personal care products and cosmetics, such as sunscreen or toothpaste, as well as to food products (e.g. chewing gums and sweets)^21^, which are sources of titanium exposure unrelated to pediatric orthopedic titanium implants. A few authors tried to determine the range of “normal” titanium blood levels to establish a threshold value for titanium-induced implant failure^22^. However, individual ranges are wide and different methodological approaches to measure metals in blood rarely give the same values for the same sample.

Reliable methods to measure titanium in biological fluids are scarce. The most commonly used approaches are ICP-MS-based techniques. However, these methods require well-trained operators and have high running costs. Additionally, comparisons across laboratories are challenging, mainly due to lack of standardized sample preparation, instrument type and settings and analytical approach^23^. Therefore, absolute titanium blood values should be interpreted carefully.

To our knowledge, this is the first study to determine the volume of abrased material from MCGRs. Biases of visual scoring were excluded and we could determine width and depth of notches, and thereby volume of abrased material, by great precision (resolution in z 0.8 nm, measuring point density was set at one measuring point per 0.25 µm). Measuring tactile traces furthermore revealed a rotating structure on the surface of the material that is invisible to the eye. In some areas, material appeared shiny, suggesting abrasion in form of a notch by visual impression, however the tactile trace revealed no notches, but only superficial abrasion of the rotating structure.

It was not possible to record some small marks of abrasion directly at the edges of individual segments, which occurred on some rods, therefore total abrasion from the segments may be slightly higher than our measured values. The rather regular patterns of notches imply that movement of the inner and outer cylinder against each other cause abrasion during the wear time in a certain position, which changes upon regular lengthening, additionally to stress during the lengthening procedure^18^. The observation that notches occur at only one or two, sometimes three adjacent segments in our study supports the hypothesis of off-axis loading causing one-sided abrasion^16^. However, further investigations with more parameters such as curve stiffness and coronal and sagittal balance are needed to define reasons for abrasion.

In our study, the leakage from the actuator of the MCGRs was not put into account as a source of titanium wear debris. However, this leakage was proven as a significant source of metallosis in a previous study where the MCGRs were cut open to allow internal components to be evaluated for metal wear^8^.

We could neither detect an influence of implantation time, number of elongations, nor of weight, height, gender or ambulatory status of the patient on metal implant abrasion or titanium plasma values. However, it cannot be excluded that influences of individual factors were overshadowed by the complex interactions of several factors for this study cohort, and that influencing factors may be found with a larger, more homogenous population.

In our study, linear regression analysis of metal abrasion and the values of titanium in plasma showed a positive correlation. Elevated levels of titanium in blood have also been observed in patients with implant failure^24^ or implant loosening^25^ and it has been proposed that titanium levels in blood, serum or plasma may be used as a biomarker for orthopedic implant performance^22,24–26^. Measurements of serum cobalt and chromium can serve as biomarker for wear of metal joint implants^27,28^ and reference levels are available for well and poorly functioning hip implants^29^. However, neither guidelines nor normal or abnormal blood values have been established for titanium, partly due to technical challenges and lacking comparability of results gained across laboratories^23^. Recently, a laboratory reference level for blood and plasma titanium in patients with well-functioning titanium hip implants has been proposed (2.2 and 2.56 µg/L for blood and plasma respectively)^30^. The authors suggested this to be a “starting point for further studies to explore the clinical usefulness of blood titanium as a biomarker of orthopedic implant performance”^30^. While technical challenges exist and elevated titanium levels may not be indicators for implant performance for all patients, it is worth further exploring the possibility of using titanium levels in plasma or other body fluids as potential biomarkers for implant performance.

The fact that we have established a protocol to measure small-scale abrasion reasonably fast and economically with our applied tactile technique, makes it appealing to use this in future larger scale studies—where potentially influencing factors for abrasion may be detected. Also, given that levels of toxicity of titanium may be understood better in the future, potential clinical application of this method may be taken into account—i.e. measuring abrasion from an implant upon removal, either instead of or additionally to measuring blood-titanium levels, may help to assess titanium load within a patient and decide about treatment with further implants.

Limitations of this study are the small sample size and even more importantly the lack of data on blood titanium values before implantation to estimate the individual increase of titanium particles in the blood. Further studies on possible transport routes of titanium ions, their distribution in organs and therapeutic approaches against spreading of titanium within the children’s body would provide better understanding of the extent and long-term effects of metal wear by implants in children.

## Conclusions

This study analyzed titanium abrasion from magnetically controlled growing rods (MCGRs), which were implanted in children for spinal deformity correction for an average of 2.6 years and correlated these findings to titanium plasma values. Using metrological techniques, titanium abrasion from MCGRs could be determined with great precision and linear regression analysis of abrasion values and the titanium plasma values determined by ICP-MS showed a positive correlation. Influencing factors on abrasion such as time of implantation, number of elongations or the patient’s ambulatory status could not be detected. Further studies are necessary to determine the effects and potential hazards of titanium in the pediatric body caused by implants treating scoliosis.

## Data Availability

All data generated or analyzed during this study are included in this published article.

## Abbreviations

MCGR: Magnetically controlled growing rod
EOS: Early-onset scoliosis
HR-ICP-MS: High-resolution inductively coupled plasma-mass spectrometer
mN: MilliNewton
